# Captive ungulates as sentinels of antimicrobial resistance: genomic and phenotypic characterization of extended-spectrum β-lactamase–producing *Escherichia coli* at a United Arab Emirates urban zoo

**DOI:** 10.3389/fvets.2026.1815262

**Published:** 2026-03-25

**Authors:** Ihab Habib, Moneeb A. Qablan, Fatema Aaref Alzaabi, Fatema Rashed Alnuaimi, Ghalya Ahmed Almemari, Glindya Bhagya Lakshmi, Safeya Al Naqbi, Fatima Al Shamsi, Dhanya Vijay, John Mhongovoyo, Sultan Khalifa Alkaabi, Amna Mana Alotaiba

**Affiliations:** 1 Department of Veterinary Medicine, College of Agriculture and Veterinary Medicine, United Arab Emirates University, Al Ain, United Arab Emirates; 2 ASPIRE Research Institute for Food Security in the Drylands (ARIFSID), United Arab Emirates University, Al Ain, United Arab Emirates; 3 Al-Ain Zoo, Al Ain, United Arab Emirates

**Keywords:** ESBL *Escherichia coli*, one health, ungulates, United Arab Emirates, whole-genome sequencing

## Abstract

Antimicrobial resistance (AMR) in captive wildlife remains poorly characterized, particularly in zoological collections within the Middle East. This study investigated the occurrence, phenotypic resistance patterns, and genomic characteristics of extended-spectrum *β*-lactamase (ESBL)–producing *Escherichia coli* in clinically healthy captive ungulates at Al Ain Zoo, one of the largest zoological institutions located in an urban area in the United Arab Emirates (UAE). Fecal samples were collected non-invasively from 101 ungulates during routine husbandry and screened for ESBL-producing *E. coli*. Antimicrobial susceptibility testing (AST) was performed using the VITEK 2 system, and the identified ESBL-producing and multidrug resistant *E. coli* isolates underwent whole-genome sequencing for resistome, virulome, plasmid, and phylogenetic analyses. ESBL-producing *E. coli* were detected in 35 of 101 animals, corresponding to a carriage frequency of 34.7% (95% CI: 25.4–43.9%). Phenotypically, all isolates were resistant to *β*-lactam antibiotics, while resistance to fluoroquinolones and tetracycline was common. In contrast, susceptibility to aminoglycosides, imipenem, and polymyxin B was fully preserved. Based on exhibiting resistance to three or more antimicrobial classes, 26 of 35 isolates (74.3%) were classified as MDR. Genomic analysis revealed that all ESBL-producing *E. coli* harbored the *bla*_CTX-M-15_ gene, frequently accompanied by resistance determinants to quinolones (*qnrS1*), tetracyclines (*tet(A)*), sulfonamides, trimethoprim, and aminoglycosides. All AMR genes were located on conjugative plasmids, predominantly IncF-type backbones. The isolates exhibited a polyclonal population structure encompassing 18 multilocus sequence types and several small SNP-defined clusters, alongside a moderate virulence gene burden lacking Shiga toxin or classical extra-intestinal pathogenic markers. This study provides the first genomic baseline of ESBL-producing *E. coli* in zoo-housed ungulates in the UAE and Middle East region, highlighting the value of urban zoological collections as sentinel sites for integrated One Health AMR surveillance supporting animal health, conservation, and public health.

## 1 Introduction

*Escherichia coli* is a common commensal of the human and animal gut, although certain strains cause intestinal and extra-intestinal infections (1). Throughout many decades, *E. coli* has accumulated numerous antimicrobial resistance genes through horizontal gene transfer mediated by plasmids and other mobile genetic elements, thereby positioning it as a widely used sentinel organism for monitoring antimicrobial resistance (AMR) in fecal bacteria across diverse hosts (2). Of particular One Health concern are extended-spectrum *β*-lactamase (ESBL)–producing *E. coli*, which are resistant to critically important third-generation cephalosporins (3GCs) and circulate across the human–animal–environment interface (3). Their presence in captive and managed animal populations underscores the value of zoos as key surveillance points for tracking antimicrobial resistance across sectors (4).

In captive zoo animals, *E. coli* with resistance to critically important antimicrobials has been well documented (5–7). A molecular survey conducted in an inner-city zoo detected 3GC-resistant *E. coli* in fecal samples from a wide range of captive mammalian species, with resistant isolates representing a substantial proportion of the cultured *E. coli* population and dominated by ESBL phenotypes, especially CTX-M–type enzymes, which are classified by the World Health Organization as critically important to human medicine (5). Similarly, studies of captive non-human primates reported high carriage rates of AMR genes, including *β*-lactamase genes, tetracycline resistance genes, and class 1 integrons, indicating an enhanced potential for horizontal gene transfer and persistence of multidrug resistance (MDR) within captive collections (6, 7). From a One Health perspective, the presence of ESBL-producing and MDR *E. coli* in zoo animals raises direct concerns for keepers, veterinarians, and animal-care workers, who experience repeated occupational exposure through animal handling, enclosure cleaning, and environmental contact (7). At the same time, AMR *E. coli* threatens animal health and conservation by complicating treatment in endangered species and facilitating the persistence and spread of resistant strains across wildlife, humans, and the environment (6).

Ungulates have substantial ecological, and cultural importance in the United Arab Emirates (UAE), and zoological institutions play a pivotal role in their conservation, captive breeding, and public education, particularly for endangered native species such as the Arabian Oryx (*Oryx leucoryx*) (8, 9). Despite their importance, AMR in zoo-housed ungulates remains poorly studied in the UAE, and worldwide, with very limited data available on both phenotypic resistance profiles and molecular characteristics of *E. coli* in these species. Addressing this gap is critical not only for safeguarding animal health and conservation outcomes, but also for strengthening One Health surveillance across the UAE and the wider Gulf Cooperation Council (GCC) region, where ungulates hold enduring cultural significance and are central to regional biodiversity and ecosystem stability (9).

The present study was conducted at Al Ain Zoo, one of the largest and most prominent zoological institutions in the UAE (10), to investigate carriage of *E. coli* in a healthy population of captive ungulates. The study aimed to comprehensively assess AMR by combining phenotypic antimicrobial susceptibility testing (AST) with genomic characterization. Specifically, a collection of ESBL-producing and MDR *E. coli* isolates were analyzed for multilocus sequence types (MLSTs), virulence and resistance gene profiles, plasmid replicon types, and core genome single-nucleotide polymorphism (SNP)–based phylogeny, providing an integrated overview of their diversity and AMR potential. This approach offers the first baseline data in the UAE and Middle East for understanding AMR dynamics in captive ungulates and supports the development of evidence-based One Health surveillance and conservation strategies in the region.

## 2 Materials and methods

### 2.1 Ethical statement

No animal ethics approval was required for this study because freshly voided fecal samples were collected (non-invasively) by zookeepers from enclosure floors during routine husbandry activities, with no direct animal handling or experimental interventions involved.

### 2.2 Study animals and sampling

Animals were selected using a convenience sampling approach integrated with routine husbandry practices and animal welfare considerations at zoological setting (11). Sampling was conducted at Al Ain Zoo; widely recognized as the largest zoo in the UAE and the entire GCC region, covering about 900–990 acres (10). The zoo where sampling took place is located in an urban setting in Al Ain City, eastern UAE, and houses an extensive collection of native and exotic species while serving as a major regional center for wildlife conservation, captive breeding, and public education (10). Sampling focused on clinically healthy captive ungulates that were accessible during daily management activities, thereby minimizing animal stress and avoiding invasive handling procedures.

A total of 101 animals were sampled (out of approximately 350 individuals belonging to the species groups presented in Table 1), representing the maximum feasible number of individuals that could be included during the study period and ensuring coverage of some of the major ungulate species of conservation importance maintained at the zoo (Table 1). Freshly voided fecal samples were collected from animals housed either individually or in stable social groups. To avoid misattribution, samples were obtained only immediately after defecation, with animals visually identified by trained zookeepers during routine morning husbandry. Collection was performed using disposable gloves and sterile tools, and each sample was placed into individually labeled sterile containers to minimize environmental cross-contamination. Sampling was conducted over a three-month period (September–November 2025). Each sample was placed into a sterile, labeled container, stored at 4 °C, and transported on the same day to the Veterinary Public Health Research Laboratory, United Arab Emirates University, for microbiological analysis. All samples were processed within 24 h of collection.

**Table 1:** Species composition and distribution of captive ungulates sampled at Al Ain Zoo, United Arab Emirates (*n* = 101).

Animal (common name)	Scientific name	Number (n)	Percentage (%)
Arabian oryx	*Oryx leucoryx*	18	17.9
Scimitar-horned oryx	*Oryx dammah*	18	17.9
Addax	*Addax nasomaculatus*	21	20.8
Common eland	*Taurotragus oryx*	16	15.8
Mhorr (dama) gazelle	*Nanger dama mhorr*	16	15.8
Dama gazelle (Dana gazelle)	*Nanger dama*	9	8.9
Beisa oryx	*Oryx beisa*	3	2.9
Total		101	100

### 2.3 Isolation and identification of ESBL-producing *Escherichia coli*

Isolation of ESBL–producing *E. coli* was carried out following the methodology described by Habib et al. (12) with minor adaptations. Briefly, approximately up to 5 g of fecal sample (with each sample corresponding to an independent animal subject (e.g., no pooling)) was aseptically inoculated into buffered peptone water (BPW; Oxoid, Basingstoke, UK) at a 1:9 (w/v) ratio and incubated at 37 °C for 18–24 h for non-selective enrichment. Following incubation, a loopful of the enriched culture was streaked onto CHROMagar™ ESBL (CHROMagar, Paris, France) and incubated at 37 °C for 24 h. Colonies exhibiting morphology and coloration consistent with presumptive ESBL-producing *E. coli* were subcultured for purification on Nutrient Agar (BioLife Italiana S.r.l., Milan, Italy) plates (12). Presumptive *E. coli* isolates were identified to the species level using matrix-assisted laser desorption/ionization time-of-flight mass spectrometry (MALDI-TOF MS; Autof MS1000, Autobio Diagnostics, China) in accordance with the manufacturer’s instructions.

### 2.4 Antimicrobial susceptibility testing (AST)

AST was performed using the VITEK 2 Compact automated system (bioMérieux, Marcy l’Étoile, France) following the manufacturer’s instructions. Testing was conducted using the AST-GN96 card, and minimum inhibitory concentration (MIC) values were generated automatically by the VITEK 2 software. The used card panel (AST-GN96) includes cephalosporins tested alone and in combination with clavulanic acid, enabling phenotypic detection ESBL production based on the observation of clavulanate synergy. MIC interpretations were reviewed and interpreted according to the Clinical and Laboratory Standards Institute (CLSI) performance standards (13). The AST panel comprised 17 antimicrobial agents representing multiple antimicrobial classes commonly used in veterinary medicine, including β-lactams (ampicillin, amoxicillin–clavulanic acid, ticarcillin–clavulanic acid, cefalexin, cefalotin, cefoperazone, ceftiofur, cefquinome, imipenem), aminoglycosides (gentamicin, neomycin), fluoroquinolones and quinolones (enrofloxacin, marbofloxacin, flumequine), tetracyclines (tetracycline), phenicols (florfenicol), and folate pathway inhibitors (trimethoprim–sulfamethoxazole). Isolates were classified as MDR if they exhibited non-susceptibility to at least one agent in three or more antimicrobial classes, in accordance with internationally recommended criteria (14). A quality control strain (*E. coli* ATCC 25922) was included during AST to ensure the accuracy and reliability of the results.

### 2.5 Short-read sequencing and bioinformatics

A subset (~85%) of ESBL–producing *E. coli* isolates recovered from captive ungulates in this study was selected for whole-genome sequencing (WGS). A small number of isolates were not included in downstream analyses because they could not be recovered after storage at −80 °C. Genomic DNA was extracted using the DNeasy Blood and Tissue Kit (Qiagen, Hilden, Germany) according to the manufacturer’s instructions. DNA concentration and purity were assessed using a NanoDrop™ spectrophotometer (Thermo Fisher Scientific, Waltham, MA, USA) and a Quantus™ Fluorometer (Promega, Madison, WI, USA). Purified DNA samples were shipped to Novogene Europe Genomics (Cambridge, United Kingdom) for Illumina short-read sequencing. Libraries were prepared using the Illumina DNA Prep Kit (Illumina Inc., San Diego, CA, USA) and sequenced on an Illumina NovaSeq platform to generate paired-end reads. Key sequencing quality metrics for the isolates examined in this study are presented in Supplementary Table 1. The sequencing data and assembled genomes generated in this study have been publicly deposited in the NCBI database under BioProject accession number PRJNA1414479.

Raw FASTQ files were uploaded to the Solu (version 1.0.485) Genomics, a cloud-based bacterial genomics platform (https://www.solugenomics.com), for comprehensive genomic characterization (15). The Solu Genomics workflow integrates validated bioinformatics pipelines for genome assembly, species confirmation, molecular typing, AMR, plasmid characterization, virulence profiling, and phylogenetic analysis. Species identification was confirmed using BactInspector v0.1.3, while MLST characterization was performed using mlst v2.23.0 with allele assignments queried against the PubMLST database (16, 17). AMR genes and resistance-associated point mutations were identified using AMRFinderPlus v3.11 (18), applying default thresholds. Virulence-associated genes were screened using ABRicate v1.0.1 against the Virulence Factor Database (VFDB) (last updated June 3, 2025) (19). Plasmid reconstruction and typing were conducted using MOB-suite v3.1.9 (20).

Phylogenetic relationships were inferred using the Solu reference-based phylogeny pipeline (v1.2.7). Briefly, sequencing reads were aligned to an appropriate reference genome using Snippy v4.6.0, followed by filtering of low-quality SNPs using an in-house script. Pairwise SNP distances were calculated using snp-dists v0.8.2, and isolates were clustered using a 20-SNP single-linkage threshold (15). The 20-SNP threshold is a commonly used starting point for investigating clonal relatedness (21, 22). It’s designed to be inclusive enough to account for sequencing errors and within-host diversity, while still excluding isolates that are too distant to be plausibly related (21, 22). Maximum-likelihood phylogenetic trees were constructed using IQ-TREE v2.3.6 (23).

## 3 Results

### 3.1 Overall detection of ESBL-producing *Escherichia coli*

ESBL-producing *E. coli* were detected in fecal samples collected from clinically healthy captive ungulates at Al Ain Zoo. Out of the 101 animals sampled, 35 yielded ESBL-producing *E. coli*, corresponding to a detection frequency of 34.7% (35/101), with a 95% confidence interval (CI) of 25.4–43.9%. These 35 ESBL-producing isolates were subsequently included in antimicrobial susceptibility testing. Of the 35 ESBL-producing *E. coli* isolates recovered, 30 (85%) were successfully subjected to whole-genome sequencing, as five isolates could not be revived after storage at −80 °C.

### 3.2 Patterns of antimicrobial sensitivity testing

**Figure 1:**
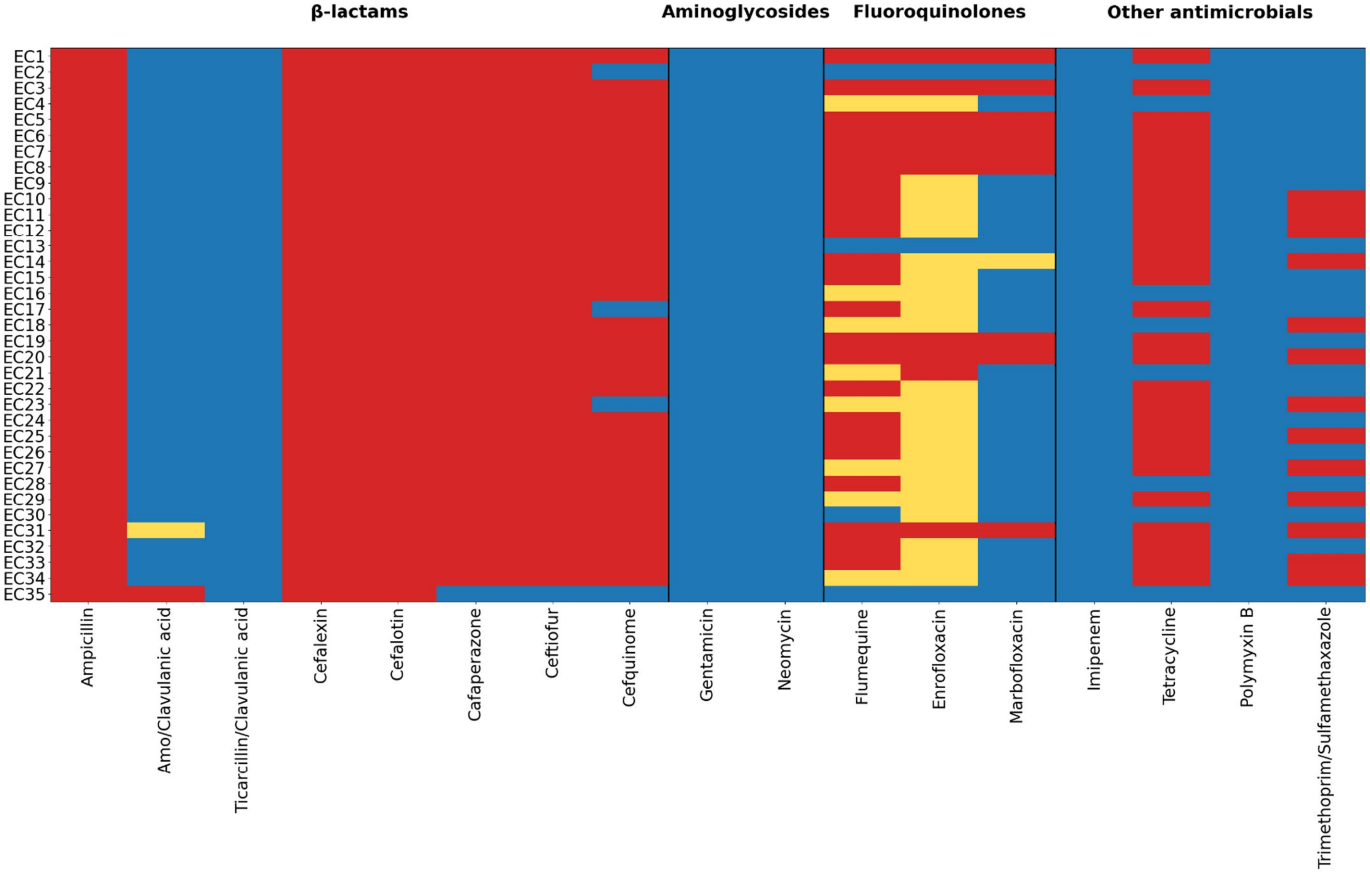
Antimicrobial susceptibility profiles of 35 ESBL-producing *Escherichia coli* isolates recovered from captive ungulates at Al Ain Zoo, United Arab Emirates. Rows represent individual isolates and columns represent antimicrobial agents grouped by antimicrobial class. Susceptibility categories are indicated by color: resistant (red), intermediate (yellow), and susceptible (blue). Vertical lines delineate antimicrobial classes.

Phenotypic antimicrobial susceptibility testing of the 35 ESBL-producing *E. coli* isolates showed uniform resistance to β-lactam antibiotics, including ampicillin and cephalosporins, with 35/35 isolates (100%) resistant to ampicillin and first-generation cephalosporins (cefalexin and cefalotin) and the majority resistant to higher-generation cephalosporins (Figure 1). Resistance to fluoroquinolones was more variable: resistance to flumequine was observed in approximately two-thirds of isolates, while resistance or intermediate susceptibility to enrofloxacin and marbofloxacin occurred in a substantial subset of isolates, as illustrated in Figure 1.

Susceptibility to aminoglycosides was largely preserved, with 35/35 isolates (100%) susceptible to gentamicin and neomycin. Among the other antimicrobial agents, all isolates (35/35; 100%) were susceptible to imipenem, and no resistance to polymyxin B was detected (Figure 1). In contrast, resistance to tetracycline was frequently observed, affecting more than half of the isolates, while susceptibility to trimethoprim–sulfamethoxazole varied, with both susceptible and resistant phenotypes present, as demonstrated in Figure 1. Applying the predefined criterion of resistance to at least one agent in ≥ 3 antimicrobial classes, 26 of the 35 isolates (74.3%) were classified as MDR (Figure 1).

### 3.3 Distribution of AMR determinants in ESBL-producing *Escherichia coli*

Sequencing generated approximately 450–600 Mbp of raw data per isolate, which was assembled into genomes of ~4.8–5.2 Mbp, with N50 values ranging from ~150–220 kbp, assembled in ~80–100 contigs, and achieving an average coverage of ~120 × (details of sequencing quality matrices for each examined isolate are given in Supplementary Table 1).

**Figure 2:**
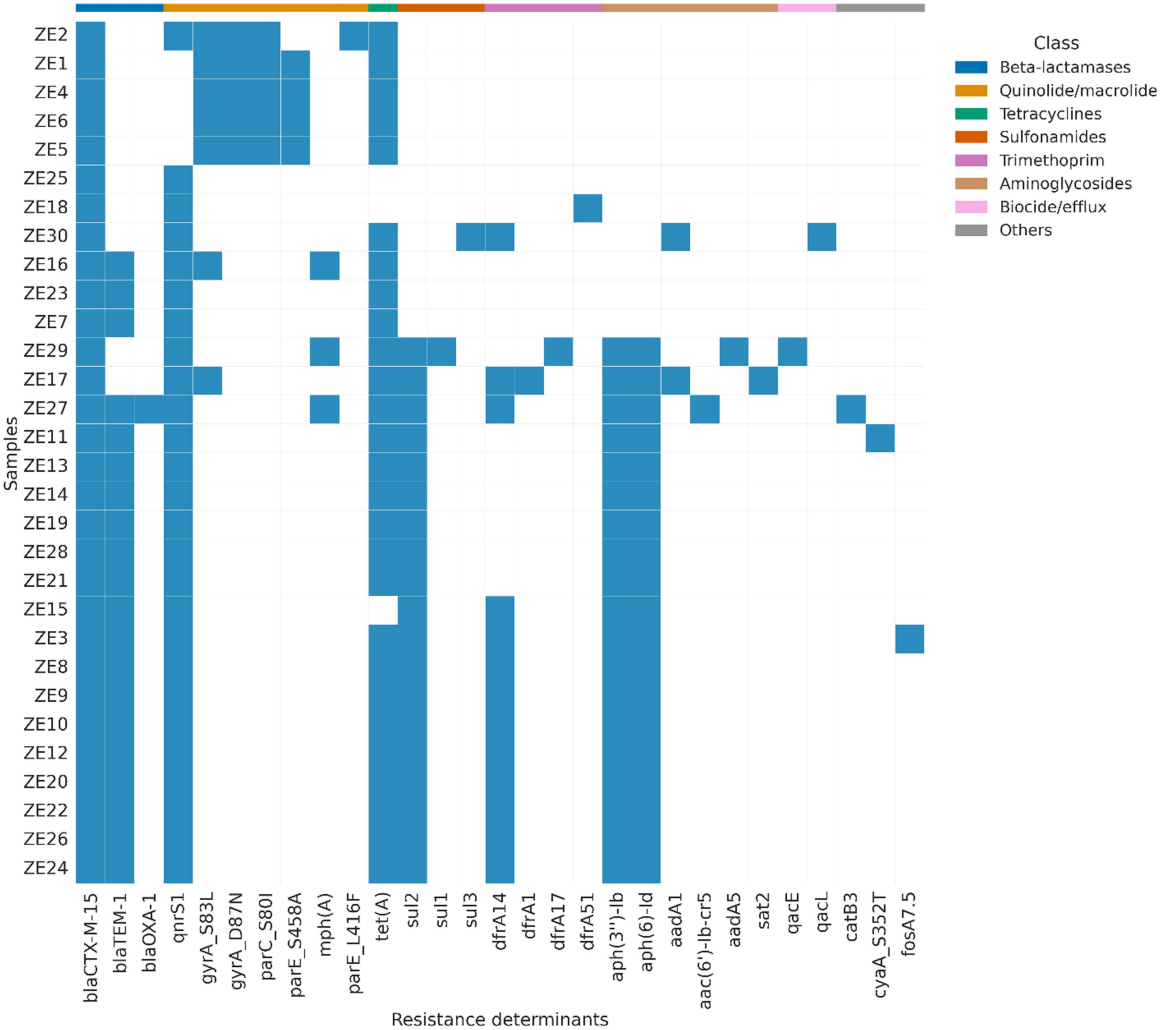
Patterns of antimicrobial resistance determinants among whole-genome sequenced ESBL-producing *Escherichia coli* isolated from captive ungulates at Al Ain Zoo, United Arab Emirates. Rows represent individual isolates and columns represent resistance determinants, grouped by antimicrobial class. Cells indicate absence (white) or presence (blue) of each determinant.

As shown in Figure 2, the ESBL gene *bla*_CTX-M-15_ was detected in all isolates (30/30; 100%), representing the dominant β-lactamase determinant in the collection. Additional β-lactamase genes were also observed, including *bla*_TEM-1_ in 20 isolates (66.7%) and *bla*_OXA-1_ in one isolate (3.3%) (Figure 2). Resistance determinants associated with quinolone resistance were frequently identified.

The plasmid-mediated quinolone resistance gene *qnrS1* was present in 26 isolates (86.7%), while chromosomal quinolone resistance–associated mutations were detected at lower frequencies, including *gyrA_S83L* in seven isolates (23.3%), *gyrA_D87N* in five isolates (16.7%), and *parC_S80I in* five isolates (16.7%). Additional mutations in *parE* (*parE_S458A and parE_L416F*) were detected in four (13.3%) and one (3.3%) isolates, respectively (Figure 2).

Genes conferring resistance to tetracyclines, predominantly *tet(A)*, were detected in 27 isolates (90.0%). Sulfonamide resistance genes were also common, with *sul2* present in 19 isolates (63.3%), while *sul1* and *sul3* were each detected in one isolate (3.3%). Trimethoprim resistance genes, including *dfrA14*, *dfrA17*, *dfrA51*, and *dfrA1*, were identified collectively in 16 isolates (53.3%), with *dfrA14* being the most frequent (13 isolates; 43.3%) (Figure 2).

Aminoglycoside resistance genes were detected in a substantial proportion of isolates, including *aph(3′′)-Ib* and *aph(6)-Id*, each present in 19 isolates (63.3%), while *aadA1* and *aadA5* were detected in two (6.7%) and one (3.3%) isolates, respectively. Genes associated with biocide resistance and efflux, including *qacE* and *qacL*, were each detected in one isolate (3.3%). Other resistance determinants, such as *catB3*, *fosA7.5*, *sat2*, and *aac(6′)-Ib-cr*, were detected at low frequencies (≤3.3%). The distribution and co-occurrence patterns of these resistance determinants across isolates are shown in Figure 2.

### 3.4 Genomic characteristics of AMR-associated conjugative plasmids

Analysis of plasmid content using MOB-suite identified AMR genes exclusively on conjugative plasmids, while no AMR genes were detected on mobilizable plasmids among the ESBL-producing *E. coli* isolates sequenced in this study. Accordingly, the analysis focused on conjugative plasmids, which are recognized as key drivers of AMR dissemination (Table 2).

**Table 2:** Genomic characteristics of antimicrobial resistance–associated conjugative plasmids identified in ESBL-producing *Escherichia coli* isolated from captive ungulates at Al Ain Zoo.

Sample name	MLST	Plasmid size (bp)	MOB Cluster	AMR genes (number, N; and identification)	Replicon types	Relaxase types	mpf
ZS30	10	162,397	AA179	*N* = 3; *dfrA14, qnrS1, tet(A)*	IncFIB, IncFIC, IncFII	MOBF, MOBP	MPF_F
ZS29	9,543	126,631	AA282	*N* = 11; *aadA5, aph(3″)-Ib, aph(6)-Id, bla*_CTX-M-15_*, dfrA17, mph(A), qacE, qnrS1, sul1, sul2, tet(A)*	IncK2/Z	MOBP	MPF_I
ZS26	58	81,615	AA887	*N* = 8; *aph(3″)-Ib, aph(6)-Id, bla*_CTX-M-15_*, bla*_TEM-1_*, dfrA14, qnrS1, sul2, tet(A)*	IncFIB	MOBC	MPF_T
ZS25	196	99,958	AA474	*N* = 2; *bla*_CTX-M-15_*, qnrS1*	IncI-gamma/K1	MOBP	MPF_I
ZE24	58	81,665	AA887	*N* = 8; *aph(3″)-Ib, aph(6)-Id, bla*_CTX-M-15_*, bla*_TEM-1_*, dfrA14, qnrS1, sul2, tet(A)*	IncFIB	MOBC	MPF_T
ZS22	48	95,999	AA887	*N* = 8; *aph(3″)-Ib, aph(6)-Id, bla*_CTX-M-15_*, bla*_TEM-1_*, dfrA14, qnrS1, sul2, tet(A)*	IncFIB	MOBC	MPF_T
ZS16	3,076	163,446	AA179	*N* = 2; *bla*_CTX-M-15_*, bla*_TEM-1_*, qnrS1, tet(A)*	IncFIB, IncFIC, IncFII	MOBF, MOBP	MPF_F
ZE14	58	71,188	AA887	*N* = 7; *aph(3″)-Ib, aph(6)-Id, bla*_CTX-M-15_*, bla*_TEM-1_*, qnrS1, sul2, tet(A)*	IncFIB	MOBC	MPF_T
ZS13	58	71,108	AA887	*N* = 7; *aph(3″)-Ib, aph(6)-Id, bla*_CTX-M-15_*, bla*_TEM-1_*, qnrS1, sul2, tet(A)*	IncFIB	MOBC	MPF_T

Plasmids were characterized using MOB-suite, including replicon type, relaxase, mating pair formation (MPF) system, predicted mobility, and associated antimicrobial resistance genes.

A total of nine AMR-associated conjugative plasmids were identified across isolates belonging to multiple MLSTs, with plasmid sizes ranging from 71,108 bp to 163,446 bp. These plasmids belonged to several MOB clusters (AA179, AA282, AA474, and AA887) and carried between 2 and 11 AMR genes per plasmid. The most frequently observed resistance genes harbored by conjugative plasmids among the present study collection were *bla*_CTX-M-15_, *bla*_TEM-1_, *qnrS1*, *tet(A)*, *sul2*, *dfrA14*, and aminoglycoside resistance genes *aph(3′′)-Ib* and *aph(6)-Id* (Table 2).

As indicated in Table 2, the identified conjugative plasmids were associated with diverse replicon types, including IncFIB, IncFIC, IncFII, IncK2/Z, and IncI-*γ*/K1. Several closely related IncFIB plasmids (MOB cluster AA887) were detected in multiple isolates of ST58 and ST48, carrying similar combinations of ESBL and companion resistance genes. Overall, these findings demonstrate that, within this ESBL-producing *E. coli* collection from captive ungulates, AMR genes were confined to conjugative plasmids with diverse backbones and gene contents, as summarized in Table 2.

### 3.5 Virulence and tolerance-associated genes

**Figure 3:**
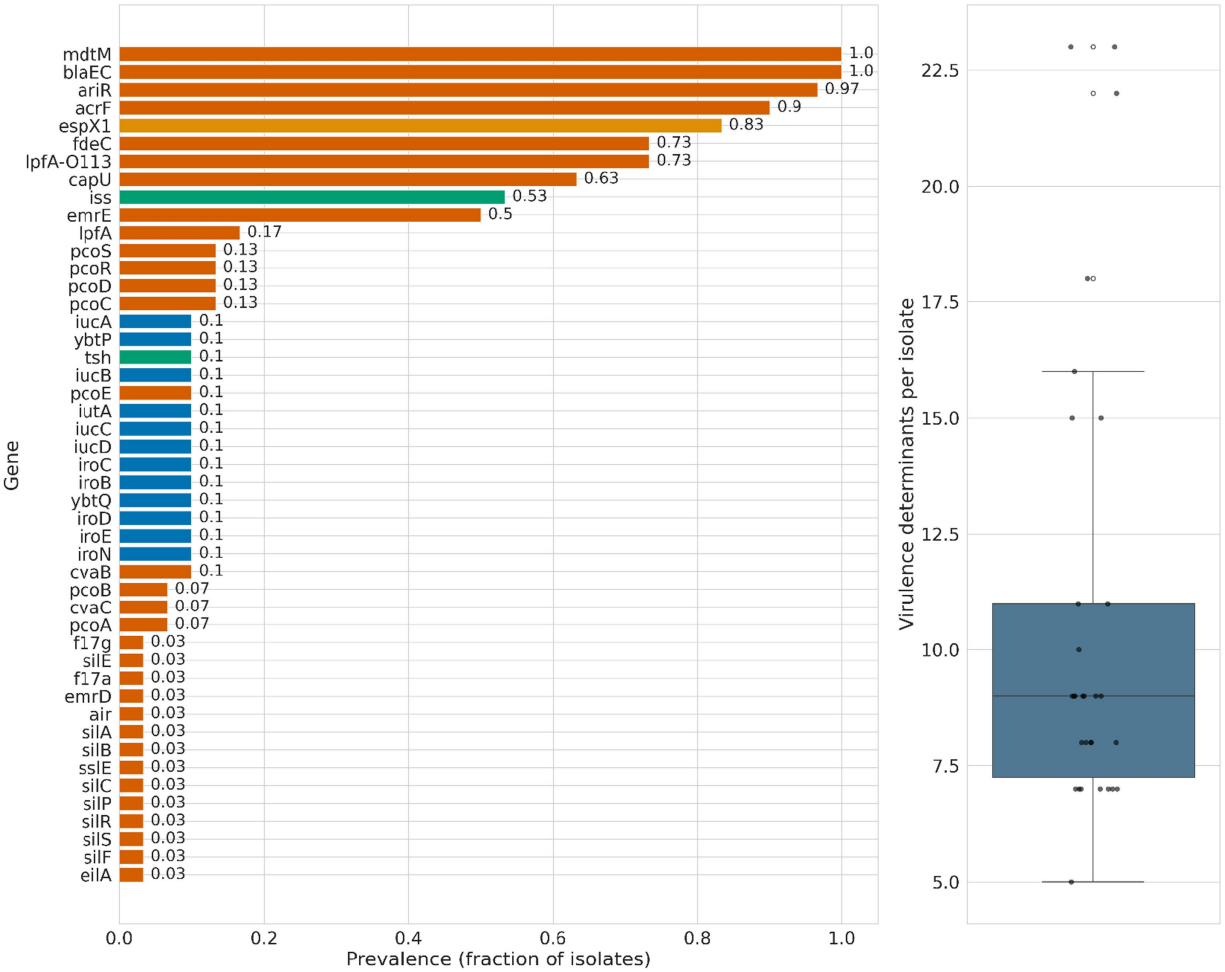
Virulence determinants identified in ESBL-producing *Escherichia coli* isolated from captive ungulates at Al Ain Zoo, United Arab Emirates. The left panel shows the prevalence of virulence genes across isolates, while the right panel summarizes the distribution of total virulence gene counts per isolate.

WGS identified a range of virulence-associated genes with variable prevalence among ESBL-producing *E. coli* isolates recovered from captive ungulates (Figure 3). Several determinants were highly prevalent, including *mdtM* (multidrug efflux transporter) (100%), followed by *ariR* (biofilm formation and stress response regulator) in 29/30 (96.7%) and *acrF* (RND family efflux pump component) in 27/30 (90.0%). Adhesion- and colonization-associated genes were also common, including *espX1* (type III secretion–associated effector) in 25/30 (83.3%), *fdeC* (intimin-like adhesin) in 22/30 (73.3%), and *lpfA-O113* (long polar fimbriae) in 22/30 (73.3%).

Additional virulence determinants were detected at moderate frequencies, including *capU* (capsule biosynthesis–associated protein) in 19/30 (63.3%), *iss* (increased serum survival) in 16/30 (53.3%), and *emrE* (small multidrug resistance efflux pump) in 15/30 (50.0%), while the remaining virulence genes occurred at lower frequencies (≤ 20%) (Figure 3, left panel). No Shiga toxin genes (*stx1* or *stx2*) were detected in any isolate, nor any classical extraintestinal pathogenic *E. coli* (ExPEC) virulence markers, such as *pap*, *sfa*, *afa*, or *cnf1*, were identified (although *iss* was present in a subset of isolates). The total virulence gene burden per isolate ranged from approximately 5 to 23 genes, with a median of ~9 virulence determinants per isolate, as shown in Figure 3 (right panel).

### 3.6 Clonal population structure and genomic diversity of ESBL-producing *Escherichia coli*

**Figure 4:**
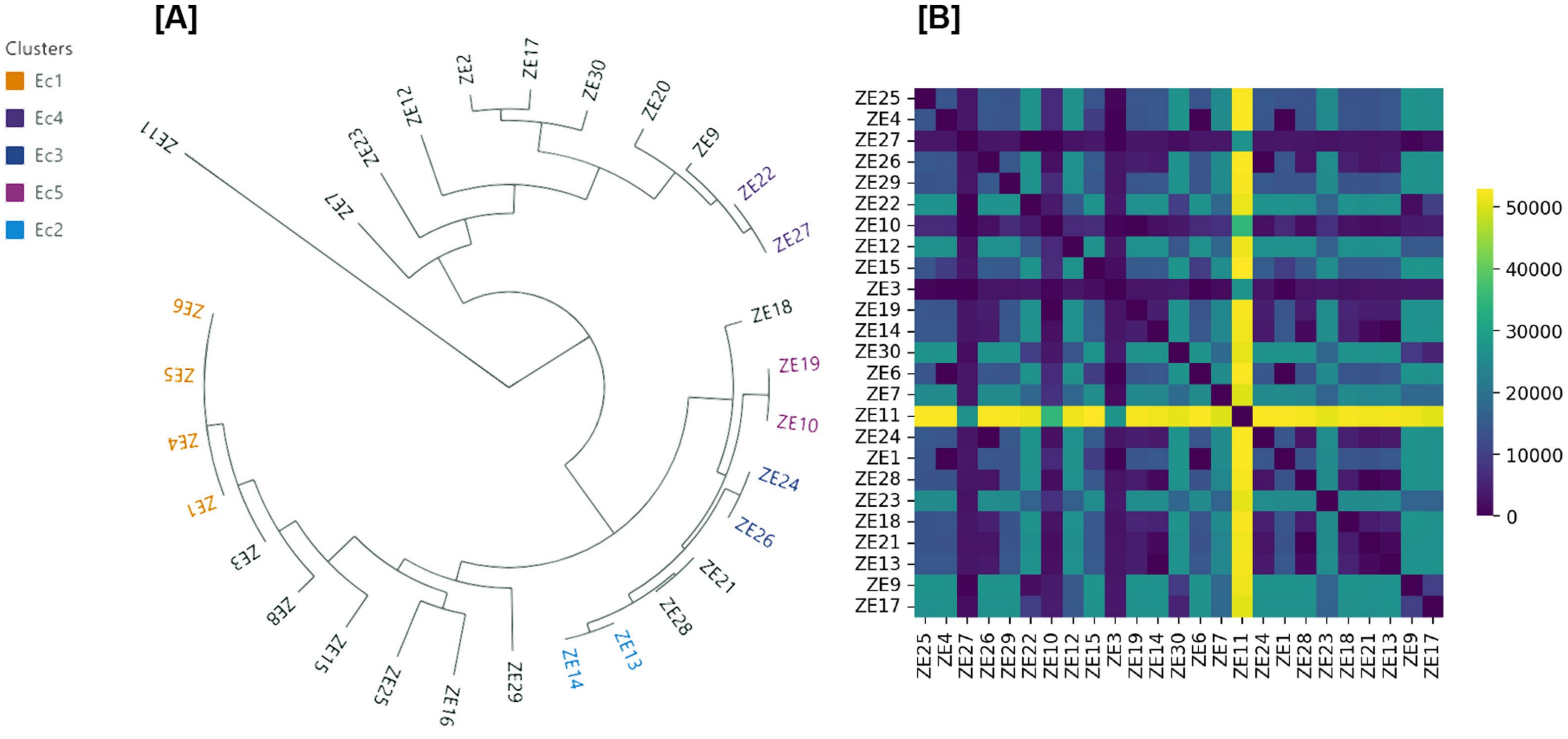
**(A)** Midpoint-rooted maximum-likelihood phylogenetic tree of ESBL-producing *Escherichia coli* isolates recovered from captive ungulates at Al Ain Zoo, United Arab Emirates. The tree was constructed based on core-genome single-nucleotide polymorphisms (SNPs) and rooted using *E. coli* K-12 substr. MG1655 as a reference genome. **(B)** Pairwise SNP differences among the genome sequenced isolates, illustrating genomic relatedness within the ungulate-associated *E. coli* population.

WGS of ESBL-producing *E. coli* isolates revealed a heterogeneous clonal population structure (Figure 4). A total of 18 distinct MLSTs were identified among the 30 sequenced isolates; the most frequently detected lineage was ST58 (7/30; 23.3%), followed by ST1431 (4/30; 13.3%) and ST10 (3/30; 10.0%), while the remaining MLSTs were represented by one or two isolates each, consistent with a predominantly polyclonal population structure (Figure 4). Pairwise SNP distance analysis further supported this population structure, with markedly higher SNP distances (>20,000 SNPs) between clusters and unrelated lineages (Figure 4, right panel).

Core-genome SNP–based phylogenetic analysis resolved the isolates into several discrete genomic clusters (Ec1–Ec5) alongside genetically distinct singletons (Figure 4, left panel). Isolates within the same cluster differed by ≤20 SNPs, indicating close genomic relatedness, while isolates belonging to different clusters or distinct MLSTs were separated by substantially larger SNP distances. Cluster Ec1 comprised 4 isolates, whereas clusters Ec2, Ec3, Ec4, and Ec5 each comprised 2 isolates, with the remaining isolates occurring as singletons. Together, these analyses demonstrate a population structure characterized by small, closely related clonal clusters embedded within a broader, genetically diverse population of ESBL-producing *E. coli* circulating among captive ungulates.

## 4 Discussion

This study provides the first whole-genome–based characterization of ESBL-producing *E. coli* in captive ungulates from a zoo in the UAE, providing baseline genomic data to support evidence-based animal health management in zoological collections in the UAE and the wider Middle East region. Our findings show that healthy zoo ungulates can serve as valuable sentinels for monitoring ESBL-producing *E. coli* in an urban UAE setting, highlighting the important role of zoological collections in One Health surveillance and proactive animal health management.

The present work focuses specifically on ungulates, a taxonomic and husbandry group that can make up a large proportion of collections in Middle Eastern zoos (8, 9), yet remains under-represented in the AMR literature regionally and internationally. The ESBL-producing *E. coli* detection frequency of 34.7% observed here is substantial for a cohort of clinically healthy ungulates sampled under routine husbandry. Comparable or higher prevalences have been reported in other zoo mammal collections. In a nationwide study of wild ungulates in Portugal (from 181 animals sampled) the researchers found ~40% (*n* = 59) of *Enterobacterales* as MDR; with CTX-M ESBLs detected included CTX-M-14, CTX-M-15 and CTX-M-98 (24). In two Belgian zoos, broad-spectrum *β*-lactamase–producing *Enterobacteriaceae* was detected in 52.6% of zoo mammals, with most isolates identified as *E. coli* and 64% showing MDR profiles (25). In Pakistan, a study detected ESBL-producing *E. coli* in 52% of fecal samples from zoo animals (26). Our study concluded prevalence therefore lies within the range of zoo-based studies from other regions, despite differences in animal taxa, sampling frames, and methodologies. By restricting the sampling frame to ungulates, our data provide the first targeted baseline for this group in the UAE and contribute to filling an acknowledged gap in zoo-focused AMR surveillance.

The phenotypic susceptibility profile observed in this study showed nearly uniform resistance to β-lactams, consistent with selection and maintenance of ESBL-producing clones under β-lactam exposure in the wider ecosystem. The MDR rate of 74.3% in our ESBL-producing isolates is high, although comparable to other zoo studies. De Witte et al. (25) reported MDR in 64% of ESBL-producing *E. coli* from Belgian zoo mammals, while a study in Pakistan reported MDR in 100% of ESBL-producing *E. coli* from zoo animals (26). In Swiss petting-zoo mammals and birds, ESBL-producing *E. coli* were also commonly MDR, frequently resisting fluoroquinolones and other critically important human medicines (27). By contrast, aminoglycosides (gentamicin, neomycin), carbapenems (imipenem) and polymyxin B retained full phenotypic activity in the isolates characterized in the present study. Such findings are encouraging from a therapeutic standpoint for high-value zoo species, particularly in cases of severe or life-threatening infections where effective antimicrobial options are limited.

Genomic analysis showed that the ESBL gene *bla*_CTX-M-15_ was detected in all examined isolates in this study, with frequent co-carriage of *bla*_TEM-1_. In the UAE, high carriage rates of *bla*_CTX-M-15_ harboring *E. coli* have recently been documented in broiler farms (28), retail meat (29), and healthy cats and dogs (30). Our findings extend this emerging national picture by showing that *bla*_CTX-M-15_ carrying ESBL-producing *E. coli* are also entrenched in captive wildlife ungulates, further emphasizing the need for integrated AMR surveillance across food animals, companion animals, wildlife and environmental compartments in the UAE. Urban zoos are particularly well suited to contribute to such efforts, as many animal species (e.g., ungulates, as the case in this study) are maintained in semi-captive, safari-style open enclosures with direct environmental exposure, positioning zoological collections as effective sentinels for systematic monitoring of AMR at the wildlife–environment interface (31).

The high prevalence of *qnrS1* and additional chromosomal QRDR mutations (*gyrA* and *parC*) provides a genomic explanation for the frequent fluoroquinolone resistance or reduced susceptibility observed phenotypically among the study isolates. Plasmid-mediated quinolone resistance genes and QRDR mutations have likewise been reported in ESBL-producing *E. coli* from captive giant pandas and non-human primates, often primarily located with ESBL genes on mobile elements (32, 33). The widespread occurrence of *tet(A)*, *sul2*, *dfrA* alleles and aminoglycoside-modifying enzymes in our isolates is also consistent with resistome profiles from ESBL-producing zoo animals in other countries (32, 33), and from broiler (34) and pet populations (30) in the UAE.

Plasmid reconstruction of the ESBL-*E. coli* collection recovered from zoo-managed ungulates in this study revealed that AMR genes were not linked to mobilizable plasmids; yet mostly confined to conjugative plasmids belonging to diverse replicon types, carrying between 2 and 11 resistance genes per plasmid. IncF-type plasmids (commonly found in this collection), in particular, are recognized as key epidemic vehicles for *bla*_CTX-M-15_ and other resistance genes in *E. coli* lineages, having co-evolved with their hosts and facilitated clonal expansion across human and animal sectors worldwide (35, 36). The detection of closely related IncFIB plasmids in multiple STs in our dataset suggests plasmid-mediated horizontal gene transfer within the zoo environment, rather than exclusively clonal spread.

In our examined collection of ESBL-*E. coli* from captive ungulates, agreement between phenotypic and genotypic resistance profiles was excellent for β-lactams and high for tetracyclines, but more modest for fluoroquinolones and poor for aminoglycosides, where aminoglycoside resistance genes were frequently detected in phenotypically susceptible isolates. Similar discordance has been noted in other WGS-based AMR studies and may reflect low or context-dependent expression of resistance genes, or mis-annotation of truncated or non-functional determinants (37). These findings underline the importance of interpreting genotypic resistance data in conjunction with phenotypic testing when making clinical decisions for individual zoo animals.

The virulence gene repertoire of the ESBL-producing *E. coli* from captive ungulates was characterized by a mixture of colonization factors (e.g., *fdeC, lpfA-O113*), stress-response and efflux systems (e.g., *mdtM, acrF, ariR, emrE*), capsule-associated genes (*capU*) and serum-resistance gene *iss*. Notably, no Shiga toxin genes (*stx1/2*) and no hallmark ExPEC adhesin/toxin genes (e.g., *pap, sfa, afa, cnf1*) were detected, suggesting that the majority of isolates represent commensal or opportunistic intestinal strains rather than pathotypes (38). Our ungulate-derived isolates therefore appear, on average, to have lower level of classical virulence potential, although the presence of *iss*, adhesins and biofilm-associated regulators indicates a capacity for persistent colonization and potential extra-intestinal infection under predisposing conditions. From a One Health perspective, the combination of high ESBL/MDR burdens with a moderate but non-negligible virulence gene load is concerning. Even if most colonizing strains do not meet strict pathotype definitions (38), their plasmid content and genomic plasticity provide a platform for acquiring additional virulence or resistance traits, especially in environments where humans, domestic animals, wildlife and environmental bacteria intersect (2).

The WGS data revealed a heterogeneous MLST distribution, despite the short window (3 months) of sampling in this study. The most frequently reported ST58 in this collection from Al Ain Zoo is increasingly recognized as a generalist lineage spanning humans, livestock, wildlife and environmental sources, often associated with ESBL production and extra-intestinal (ExPEC) infections (39), while ST10 belongs to a broad commensal/ExPEC complex common in food animals and humans (40). The presence of such globally distributed, host-generalist lineages in captive ungulates suggests that resistant *E. coli* are likely to be frequently introduced into the zoo from external reservoirs (e.g., city birds, personnel, breeding centers, or pests) and then maintained or further disseminated within the collection. Core-genome phylogeny and SNP clustering identified several small clonal clusters (≤20 SNPs apart) alongside many genetically distinct singletons, indicating that both clonal expansion between closely hosted and socially connected ungulate groups could be evident (21, 22), but also repeated introductions contribute to the genotypic diversity. Similar mixtures of clonal clusters and unrelated lineages have been documented in zoo mammals in Europe and in Mexican petting-zoo birds (25, 41), supporting the view that zoo populations act as “mixing points” for AMR strains from diverse origins.

Key strengths of this study include the focus on an under-studied host group (captive ungulates), the integration of phenotypic AST with WGS-based resistome, virulome, plasmid and phylogenetic analyses, and the positioning of the work within a major urban Middle Eastern zoo that plays a significant conservation and educational role (10). However, the sample size could have been improved, despite being comparable to earlier zoo studies elsewhere (11, 31). The convenience sampling approach adopted in our study carries additional limitation; as such an approach may not capture all species or age classes equally. The narrow sampling period in this study (three months), along with the cross-sectional design, precluded assessment of temporal dynamics or persistence within individuals. Future work should include longitudinal sampling of the same enclosures and individuals, environmental sampling (water, soil, fomites), and integrated analyses of staff and, where appropriate, visitor exposures to map transmission pathways more precisely.

## 5 Conclusion

This study shows that clinically healthy captive ungulates at a major urban zoo in the UAE harbor a diverse population of ESBL-producing *E. coli*. The examined isolates characterized by a high rate of multidrug resistance, and a resistome dominated by *bla*_CTX-M-15_ commonly carried on conjugative plasmids together with companion resistance determinants. Despite extensive *β*-lactam resistance, susceptibility to carbapenems, polymyxin B, and aminoglycosides was largely preserved. Whole-genome analysis revealed a polyclonal population structure with multiple MLST lineages, limited clonal clustering, and a moderate virulence gene burden lacking Shiga toxin or classical ExPEC markers. These findings provide the first genomic baseline for ESBL-producing *E. coli* in zoo-housed ungulates in the UAE and wider Middle East region, adding an important wildlife and conservation dimension to the regional One Health AMR landscape. Given the close interface between ungulates, veterinary staff, and the urban environment, zoos represent valuable sentinel sites for AMR surveillance. Zoo management practices should endorse high standards of hygiene and effective waste-management practices that collectively protect animal health, strengthen conservation programs, and also contribute to public health.

## Data Availability

The datasets presented in this study can be found in online repositories. The names of the repository/repositories and accession number(s) can be found at: https://www.ncbi.nlm.nih.gov/, https://www.ncbi.nlm.nih.gov/bioproject/?term=PRJNA1414479.

## References

[1] Bélanger L Garenaux A Harel J Boulianne M Nadeau E Dozois CM. *Escherichia coli* from animal reservoirs as a potential source of human extraintestinal pathogenic *E. coli*. FEMS Immunol Med Microbiol. (2011) 62:1–10. doi: 10.1111/j.1574-695X.2011.00797.x, 21362060

[2] Anjum MF Schmitt H Börjesson S Berendonk TU Donner E Stehling EG . The potential of using *E. coli* as an indicator for the surveillance of antimicrobial resistance (AMR) in the environment. Curr Opin Microbiol. (2021) 64:152–8. doi: 10.1016/j.mib.2021.09.011, 34739920

[3] Mandujano-Hernández A Martínez-Vázquez AV Paz-González AD Herrera-Mayorga V Sánchez-Sánchez M Lara-Ramírez EE . The global rise of ESBL-producing *Escherichia coli* in the livestock sector: a five-year overview. Animals. (2024) 14:2490. doi: 10.3390/ani14172490, 39272275 PMC11394230

[4] Vittecoq M Godreuil S Prugnolle F Durand P Brazier L Renaud N . Antimicrobial resistance in wildlife. J Appl Ecol. (2016) 53:519–29. doi: 10.1111/1365-2664.12596

[5] Sealey J Avison M. An oasis of resistant *Escherichia coli* in animals at an inner-city zoo. Int J Infect Dis. (2023) 130:S146. doi: 10.1016/j.ijid.2023.04.359

[6] Zhu Z Jiang S Qi M Liu H Zhang S Liu H . Prevalence and characterization of antibiotic resistance genes and integrons in *Escherichia coli* isolates from captive non-human primates of 13 zoos in China. Sci Total Environ. (2021) 798:149268. doi: 10.1016/j.scitotenv.2021.149268, 34333432

[7] Zhong W Zhou Y Che M Wang L Tian X Wang C . Extended-spectrum β-lactamase-producing *Escherichia coli* isolated from captive primates: characteristics and horizontal gene transfer ability analysis. PLoS One. (2025) 20:e0321514. doi: 10.1371/journal.pone.0321514, 40215220 PMC11990791

[8] Goursi UH Rapaie M Mehmood A. Conserving the hidden nature: an overview on conservation efforts in United Arab Emirates (UAE). Annu Res Rev Biol. (2015) 7:396–405. doi: 10.9734/ARRB/2015/18928

[9] Clarke C Alsharif SM. The lost large mammals of Arabia. J Biogeogr. (2025) 52:e15086. doi: 10.1111/jbi.15086

[10] Khaleeli M Jawabri A AlKhmeiri N. A study on visitor’s satisfaction towards services and facilities provided by Al Ain zoo. Int J Manag. (2020) 11:50–60. doi: 10.34218/IJM.11.8.2020.021

[11] Lima BP Marques AR Filho NMP de Freitas CMP Costa LN Melo LS . Prevalence and antimicrobial resistance profile of potentially zoonotic Enterobacterales isolated from macaws in zoos of northeastern Brazil. Braz J Microbiol. (2025) 56:3071–80. doi: 10.1007/s42770-025-01799-3, 41193875 PMC12660590

[12] Habib I Mohteshamuddin K Mohamed MYI Lakshmi GB Abdalla A Bakhit Ali Alkaabi A. Domestic pets in the United Arab Emirates as reservoirs for antibiotic-resistant bacteria: a comprehensive analysis of extended-spectrum beta-lactamase producing *Escherichia coli* prevalence and risk factors. Animals. (2023) 13:1587. doi: 10.3390/ani13101587, 37238016 PMC10215154

[13] Clinical and Laboratory Standards Institute (CLSI). Performance standards for antimicrobial susceptibility testing. 33rd ed. CLSI supplement M100. Wayne, PA, USA: Clinical and Laboratory Standards Institute. (2003). Available online at: https://clsi.org/media/3k3idx3b/part_c_breakpoint_implementation_summary.pdf (Accessed 16 December 2025).

[14] Magiorakos AP Srinivasan A Carey RB Carmeli Y Falagas ME Giske CG . Multidrug-resistant, extensively drug-resistant and pandrug-resistant bacteria: an international expert proposal for interim standard definitions for acquired resistance. Clin Microbiol Infect. (2012) 18:268–81. doi: 10.1111/j.1469-0691.2011.03570.x, 21793988

[15] Saratto T Visuri K Lehtinen J Ortega-Sanz I Steenwyk JL Sihvonen S. Solu: a cloud platform for real-time genomic pathogen surveillance. BMC Bioinformatics. (2025) 26:12. doi: 10.1186/s12859-024-06005-z, 39806295 PMC11731562

[16] Underwood A. BactInspector [software]. Available online at: https://gitlab.com/antunderwood/bactinspector (Accessed 17 September 2024).

[17] Seemann T. Mlst [software]. Available online at: https://github.com/tseemann/mlst (Accessed 17 September 2024).

[18] Feldgarden M Brover V Gonzalez-Escalona N Frye JG Haendiges J Haft DH . AMRFinderPlus and the reference gene Catalog facilitate examination of the genomic links among antimicrobial resistance, stress response, and virulence. Sci Rep. (2021) 11:12728. doi: 10.1038/s41598-021-91456-0, 34135355 PMC8208984

[19] Zhou S Liu B Zheng D Chen L Yang J. VFDB 2025: an integrated resource for exploring anti-virulence compounds. Nucleic Acids Res. (2025) 53:D871–7. doi: 10.1093/nar/gkae968, 39470738 PMC11701737

[20] Robertson J Nash JHE. MOB-suite: software tools for clustering, reconstruction and typing of plasmids from draft assemblies. Microb Genom. (2018) 4:e000206. doi: 10.1099/mgen.0.000206, 30052170 PMC6159552

[21] Ludden C Coll F Gouliouris T Restif O Blane B Blackwell GA . Defining nosocomial transmission of *Escherichia coli* and antimicrobial resistance genes: a genomic surveillance study. Lancet Microbe. (2021) 2:e472–80. doi: 10.1016/S2666-5247(21)00117-8, 34485958 PMC8410606

[22] Gorrie CL Da Silva AG Ingle DJ Higgs C Seemann T Stinear TP . Key parameters for genomics-based real-time detection and tracking of multidrug-resistant bacteria: a systematic analysis. Lancet Microbe. (2021) 2:e575–83. doi: 10.1016/S2666-5247(21)00149-X 35544081

[23] Minh BQ Schmidt HA Chernomor O Schrempf D Woodhams MD von Haeseler A . IQ-TREE 2: new models and efficient methods for phylogenetic inference in the genomic era. Mol Biol Evol. (2020) 37:1530–4. doi: 10.1093/molbev/msaa015, 32011700 PMC7182206

[24] Dias D Torres RT Kronvall G Fonseca C Mendo S Caetano T. Assessment of antibiotic resistance of *Escherichia coli* isolates and screening of *Salmonella* spp. in wild ungulates from Portugal. Res Microbiol. (2015) 166:584–93. doi: 10.1016/j.resmic.2015.03.006, 25869224

[25] De Witte C Vereecke N Theuns S De Ruyck C Vercammen F Bouts T . Presence of broad-spectrum beta-lactamase-producing Enterobacteriaceae in zoo mammals. Microorganisms. (2021) 9:834. doi: 10.3390/microorganisms9040834, 33919869 PMC8070755

[26] Riaz A Shahzad MA Ahsan A Aslam R Usman M Rasheed B . Investigations into the role of zoo animals in transmitting the extended spectrum beta lactamases (ESBL) *E. coli* in the environment. Int J Vet Sci. (2023) 12:832–7. doi: 10.47278/journal.ijvs/2023.046

[27] Isler M Wissmann R Morach M Zurfluh K Stephan R Nüesch-Inderbinen M. Animal petting zoos as sources of Shiga toxin-producing *Escherichia coli*, *Salmonella* and extended-spectrum β-lactamase (ESBL)-producing Enterobacteriaceae. Zoonoses Public Health. (2021) 68:79–87. doi: 10.1111/zph.12798, 33382208

[28] Khalifa HO Mohammed T Elbediwi M Abdalla A Mohamed MYI Ramadan H . Prevalence and genetic basis of extended-spectrum β-lactamase-producing *Escherichia coli* carriage in broiler farms in the United Arab Emirates. Front Vet Sci. (2025) 12:1714381. doi: 10.3389/fvets.2025.1714381, 41445590 PMC12723266

[29] Habib I Elbediwi M Mohamed MYI Ghazawi A Abdalla A Khalifa HO . Enumeration, antimicrobial resistance and genomic characterization of extended-spectrum β-lactamases producing *Escherichia coli* from supermarket chicken meat in the United Arab Emirates. Int J Food Microbiol. (2023) 398:110224. doi: 10.1016/j.ijfoodmicro.2023.110224, 37167788

[30] Habib I Elbediwi M Mohteshamuddin K Mohamed MYI Lakshmi GB Abdalla A . Genomic profiling of extended-spectrum β-lactamase-producing *Escherichia coli* from pets in the United Arab Emirates: unveiling colistin resistance mediated by *mcr-1.1* and its probable transmission from chicken meat – a one health perspective. J Infect Public Health. (2023) 16:163–71. doi: 10.1016/j.jiph.2023.10.034 37957104

[31] Frank MA Justice WS La Ragione R Chambers MA. Antibiotic resistance in *Escherichia coli* and *Enterococcus* spp. isolated from ungulates at a zoological collection in the United Kingdom. J Zoo Wildl Med. (2021) 51:761–70. doi: 10.1638/2020-0096, 33480556

[32] Wang Y He T Han J Wang J Foley SL Yang G . Prevalence of ESBLs and PMQR genes in fecal *Escherichia coli* isolated from the non-human primates in six zoos in China. Vet Microbiol. (2012) 159:53–9. doi: 10.1016/j.vetmic.2012.03.009, 22487457

[33] Wen J Okyere SK Shi Y Qu Y Chen C. Phenotypic and genotype patterns of antimicrobial resistance in non-human primates: an overlooked “one health” concern. Antibiotics. (2025) 14:985. doi: 10.3390/antibiotics14100985, 41148677 PMC12561749

[34] Habib I Mohamed MYI Lakshmi GB Ghazawi A Khan M Khalifa HO. Characterizing antimicrobial resistance and plasmidome diversity in *Escherichia coli* from imported frozen broiler chicken in the United Arab Emirates. Front Microbiol. (2025) 16:1590906. doi: 10.3389/fmicb.2025.1590906, 40589572 PMC12206764

[35] Agyekum A Fajardo-Lubián A Ansong D Partridge SR Agbenyega T Iredell JR. *bla*_CTX-M-15_ carried by IncF-type plasmids is the dominant ESBL gene in *Escherichia coli* and *Klebsiella pneumoniae* at a hospital in Ghana. Diagn Microbiol Infect Dis. (2016) 84:328–33. doi: 10.1016/j.diagmicrobio.2015.12.010, 26830052

[36] Irrgang A Falgenhauer L Fischer J Ghosh H Guiral E Guerra B . CTX-M-15-producing *E. coli* isolates from food products in Germany are mainly associated with an IncF-type plasmid and belong to two predominant clonal *E. coli* lineages. Front Microbiol. (2017) 8:2318. doi: 10.3389/fmicb.2017.02318, 29209306 PMC5702323

[37] Tyson GH McDermott PF Li C Chen Y Tadesse DA Mukherjee S . WGS accurately predicts antimicrobial resistance in *Escherichia coli*. J Antimicrob Chemother. (2015) 70:2763–9. 26142410 10.1093/jac/dkv186 PMC11606221

[38] Robins-Browne RM Holt KE Ingle DJ Hocking DM Yang J Tauschek M. Are *Escherichia coli* pathotypes still relevant in the era of whole-genome sequencing? Front Cell Infect Microbiol. (2016) 6:141. doi: 10.3389/fcimb.2016.00141, 27917373 PMC5114240

[39] McKinnon J Chowdhury PR Djordjevic SP. Genomic analysis of multidrug-resistant *Escherichia coli* ST58 causing urosepsis. Int J Antimicrob Agents. (2018) 52:430–5. doi: 10.1016/j.ijantimicag.2018.06.017, 29966679

[40] Machado MAM Panzenhagen P Lázaro C Rojas M Figueiredo EES Conte-Junior CA. Unveiling the high diversity of clones and antimicrobial resistance genes in *Escherichia coli* originating from ST10 across different ecological niches. Antibiotics. (2024) 13:737 39200037 10.3390/antibiotics13080737 PMC11350709

[41] Casas-Paul J Bravo-Ramos JL Sánchez-Otero MG Sánchez-Montes S Bonilla-Rojas S Ortíz-Carbajal LA . Presence of extended-spectrum beta-lactamase-producing *Escherichia coli* and *Klebsiella pneumoniae* isolated from avian species in a petting zoological garden. J Zool Bot Gard. (2025) 6:42. doi: 10.3390/jzbg6030042

